# Cytotoxicity of QMix™ endodontic irrigating solution on human bone marrow mesenchymal stem cells

**DOI:** 10.1186/1472-6831-14-27

**Published:** 2014-03-29

**Authors:** Ahmad AlKahtani, Sarah M Alkahtany, Amer Mahmood, Mona A Elsafadi, Abdullah M Aldahmash, Sukumaran Anil

**Affiliations:** 1 Department of Restorative Dental Sciences, College of Dentistry, King Saud University, Post Box 60169, Riyadh 11545, Saudi Arabia; 2 Stem Cell Unit, Department of Anatomy College of Medicine, King Saud University, P.O. Box 2925, Riyadh 11461, Saudi Arabia; 3 Department of Periodontics and Community Dentistry, College of Dentistry, King Saud University, Riyadh, Post Box 60169 11545, Saudi Arabia

**Keywords:** Cytotoxicity, Sodium hypochlorite, QMix™, Mesenchymal stem cells, Root canal irrigants

## Abstract

**Background:**

Debridement and disinfection of the root canal system is a crucial step in endodontic procedures. The effectiveness of irrigation relies on both the mechanical flushing action and the ability of irrigants to dissolve tissue and kill bacteria. The objective of the present study is to evaluate and compare the cytotoxicity of QMix™ root canal irrigating solution on immortalized human bone marrow mesenchymal stem cells (hTERT-MSC-C1) and to compare it with that of sodium hypochlorite (NaOCl).

**Methods:**

Immortalized human bone marrow mesenchymal stem cells (hTERT-MSCs) were exposed to QMix™ and NaOCl. Cell viability was assessed by 3-(4, 5-dimethylthiazol-2-yl)-2,5-diphenyltetrazolium bromide (MTT) and alamarBlue assays. The cell morphology was studied after two hours of exposure to QMix™ and NaOCl. Scanning electron microscopy (SEM) analyses were performed after 2- and 4-hour incubation periods. Finally, ethidium bromide/acridine orange (EB/AO) fluorescent stain was applied to the cells in the 8-chamber slides after they were incubated with the testing agents for 2 hours to detect live and dead cells. The observations were tabulated and analyzed statistically.

**Results:**

QMix™ exposure resulted in a significantly higher percentage of cell viability than NaOCl in the MTT and alamarBlue assays at three time points compared to the control. The SEM analysis demonstrated minimal morphological changes associated with cells that were exposed to the QMix™ solution, with little shrinkage and fragmentation of the cell wall. The live/dead analysis showed that the number of live cells after exposure to QMix™ was similar to that of the untreated control. No cell structure could be observed with the NaOCl group, indicating cell lysis.

**Conclusion:**

Both the QMix™ and NaOCl solutions were toxic to human bone marrow MSCs. Each solution might have induced cell death in a different way as evidenced in the cell viability, SEM and fluorescent studies. The slower cell death induced by QMix™ might therefore be less aggressive and more acceptable to living tissues.

## Background

The success of endodontic therapy depends on the eradication of microbes from the root canal system and the subsequent prevention of reinfection. Root canal irrigation has a key role in the success of endodontic treatment. During and after instrumentation, irrigants facilitate the removal of microorganisms, tissue remnants, and dentin chips from the root canal through a flushing mechanism [1,2].

An ideal root canal irrigant solution should be nontoxic, with a broad antimicrobial spectrum and the ability to dissolve necrotic pulp tissue, inactivating endotoxins, and either prevent the formation of a smear layer or dissolve it [3,4]. Currently, no single solution is able to achieve these goals, and the combined, concomitant or sequential use of two or more irrigating solutions is thus required [5]. Sodium hypochlorite (NaOCl) and chlorhexidine digluconate (CHX) are two common antibacterial agents used as root canal irrigants [5-7].

Currently, sodium hypochlorite (NaOCl) (0.5–6.15%) and EDTA (15–17%) are the two most commonly used intracanal irrigants [5,8]. Although sodium hypochlorite has most of the desirable properties, it also can produce cytotoxicity and severe inflammatory reactions [9,10]. Ethylenediaminetetraacetic acid (EDTA) is effective for removing the inorganic component of the smear layer [11].

However, due to undesirable outcomes, a combination of these irrigants is not advisable [12-15]. Hence, the sequential use of EDTA, CHX and NaOCl has been advocated by many researchers to produce optimal root canal irrigation results [16-18]. It is imperative that the root canal irrigants must not hamper the healing process of the apical region. The biocompatibility of these materials is important, as they come into contact with the peri-radicular tissues.

QMix™ is a 2-in-1 solution containing a bisbiguanide antimicrobial agent (2% CHX) and a polyaminocarboxylic acid calcium-chelating agent (17% EDTA) [19]. QMix™ was found to be effective in removing the smear layer and also has substantial antimicrobial properties [19-22]. However, data regarding the cytotoxicity of this agent are not available. Hence, the present study was conducted to evaluate and compare the cytotoxicity of the QMix™ irrigating solution on immortalized human bone marrow mesenchymal stem cells (hTERT-MSC-C1) using cell viability assays, cell morphology evaluation, and fluorescence light microscopy; 5.2% NaOCl was used for comparison.

## Methods

The study was approved by the Departmental review board of College of Dentistry Research Centre, king Saud University, Riyadh.

### Test solutions

QMix™2-in-1 (Dentsply Tulsa Dental, OK, USA) and 5.25% NaOCl (Clorox Co., Oakland, CA, USA) were used in this study.

### Cell culture

Immortalized human bone marrow mesenchymal stem cells (hTERT-MSC-C1), at the 25^th^ passage, were used for all experiments in this study. The protocol of Simbula et al. [23] was used in this study, with some modifications. The human bone marrow mesenchymal stem cells were provided by Professor Moustapha Kassem at the Odense University Hospital, Denmark [24]. The cryopreserved cells were rapidly thawed, transferred into a T-75 flask (BD Falcon™, NJ, USA) and cultured in Dulbecco’s modified Eagle’s medium (Gibco®), which was supplemented with glutamine (Gibco®), 1% penicillin-streptomycin (Gibco®), 10% fetal bovine serum (FBS Gibco®), and 1% non-essential amino acids (HyClone®), in a humidified atmosphere of 5% CO_2_/95% O_2_ at 37°C. At 70% confluence, the medium was aspirated, and the cells were washed with phosphate-buffered saline. Three milliliters of pre-warmed 0.05% trypsin/EDTA (Gibco®) was added to the flask, and the cells were incubated for 1 minute. After gentle tapping, cell detachment was checked under an inverted light microscope (Observer A1, Zeiss®, Gottingen, Germany), and 12 ml of culture media was then added to the flask to neutralize the enzymatic effect of trypsin. For cell counting, two samples (10 μl) were taken from the cell suspension after appropriate mixing. The samples were placed in the upper and lower chambers of a Neubauer hemocytometer counting chamber (Paul Marienfeld GmbH & Co. KG, Lauda-Königshofen, Germany), and the cells were counted manually under an inverted microscope (10X magnification). The cells were seeded on different plates and slides, as discussed below, for each part of the study. All procedures were performed under a class II laminar flow hood (LabGard ES 425 Biological Safety Cabinet, NuAire®).

### MTT cell viability assay

Cells were seeded in 96-well plates (clear, flat bottom, Polystyrene TC-Treated 96-well microplates) at a concentration of 1 × 10^4^ cells/well and were then incubated for 24 hours to allow cell adherence to the bottom of the wells. Culture media were then aspirated from each well and replaced with 150 μl of sterile solution (QMix™ or NaOCl). Eight wells were used as replicates for each group.

Each subgroup was incubated for the following periods: 2, 4, or 24 hours. Then, 10 μl of the MTT reagent (Cayman Chemical Company, Ann Arbor, MI, USA) was added to each well. Thereafter, 96-well plates were further incubated for 3 hours at 37°C. Finally, the solution from each well was aspirated, and 150 μl of dissolving agent was added to dissolve the formazan precipitate. The absorbance of each sample was measured with a microplate reader (Epoch Microplate Spectrophotometer, BioTek®) at a wavelength of 570 nm. The data were gathered using Gen5 Data Analysis Software (BioTek®, USA). The experiment was repeated twice for each group in each interval.

### AlamarBlue (AB) cell viability assay

The same groups that were used for the MTT assay (mentioned above) were used for the AB assay with the same intervals. The test agents were added to each well. After 2-, 4-, and 24-hour incubation periods, 10% AB reagent (Serotec®, Oxford, UK) was added to each well. After the plates were further incubated for 4 hours, the fluorescence of each well was measured at wavelengths of 530/25 and 590/35 nm excitation/emission using a fluorescence reader (BioTek®). The data were gathered using the Gen5 Data Analysis Software (BioTek®, USA). The experiment was repeated twice for each group in each interval.

### Cell morphology assessment

Cell morphology was evaluated with SEM after 2 and 4 hours of exposure to the test solutions. Briefly, 8 × 10^4^ cells/well were seeded onto glass cover slides (1 × 1.5 cm) in 6-well plates (CellStar®, Carrollton, TX) overnight. The next day, the cells were exposed to the test solutions. Two milliliters of each sterile irrigation solution was added to the slides in each well. Control untreated cells were maintained in culture medium. Immediately after the test solutions were added, the cells were examined under an inverted LM (10x magnification). At the end of the incubation periods (2 and 4 hrs), the solutions in each well were aspirated. The slides were washed with PBS and were then fixed with 2.5% glutaraldehyde in 0.1 M Na cacodylate buffer (pH 7.2) at room temperature. Thereafter, the specimens were washed with 0.1 M sodium cacodylate buffer (pH 7.2).

After fixation, the specimens were treated with 1% osmium tetroxide for 1 hour. Then, they were washed with distilled water and dehydrated using graded ethyl alcohol, in concentrations of 50%, 70%, 80%, 90% (5 minutes each), 95% (twice, 15 minutes each), and finally, 100% absolute alcohol (twice, 30 minutes each). The specimens were dried using a critical point dryer with CO_2_ (SADRI-PVT-3B). Slides were mounted on copper stubs with double adhesive tape and were then gold sputter coated to a thickness of 5–7 μm. The specimens were then observed and photographed using a JSM-6360 LV scanning electron microscope.

Two evaluators assessed the photomicrographs. The cell morphology was described according to the following criteria: the shape of the cell (normal or abnormal compared with the control), attachment to the subsurface, attachment to other cells, cytoplasmic surface extensions (blebs or microvilli), and cell wall integrity. Roundness of the cells, the presence of blebs, or detachment of the cells indicated greater cell injury [25,26].

#### Live/dead analysis

Two solutions were selected for the live/dead analysis. A modified Eagle’s medium (MEM) without phenol red (Gibco®, Gaithersburg, MD) was used for this experiment. Briefly, cells were seeded in three 8-chamber slides (Lab-Tek®) at a concentration of 1.5 × 10^4^ cells/well and were then incubated for 24 hrs to allow cell adhesion to occur. The culture medium in each well was replaced with 300 μl of each solution and then incubated for 2 hrs. Finally, 10 μl of EB/AO fluorescent dye was added to each chamber, and the fluorescence of the cells was analyzed under a fluorescent inverted microscope (ECLIPSE Ti, Nikon, Tokyo, Japan), with 10X magnification. Images were captured with imaging software (NIS-Elements, Nikon, Tokyo, Japan). The EB/AO fluorescent dye was prepared by mixing 15 mg acridine orange with 50 mg ethidium bromide powder that was first dissolved in 1 ml 95% ethanol and then diluted in 49 ml of distilled water (dH_2_O).

The images were evaluated by two evaluators according to previously described criteria. Under the green filter, an intact green nucleus, which is comparable to the control, indicates a viable cell, whereas a green fragmented nucleus indicates early apoptosis. A red nucleus indicates a ruptured cell wall, whereas a red intact nucleus indicates necrosis, and a fragmented red nucleus indicates late apoptosis under the red filter [27,28].

### Data and statistical analysis

The results of the MTT and AlamarBlue assays were calculated as percentages relative to the control (100% = no toxicity). The data were analyzed using SPSS Pc + version 21.0 statistical software. Descriptive statistics (mean and standard deviation) were used to describe continuous outcome variables. Student’s *t*-test for independent samples was used to compare the mean values of two groups. A one-way analysis of variance, followed by a multiple comparison Tukey’s test, was used to compare the mean values of the three groups. A p-value of < = 0.05 was considered statistically significant.

## Results

### MTT assay

**Figure 1:**
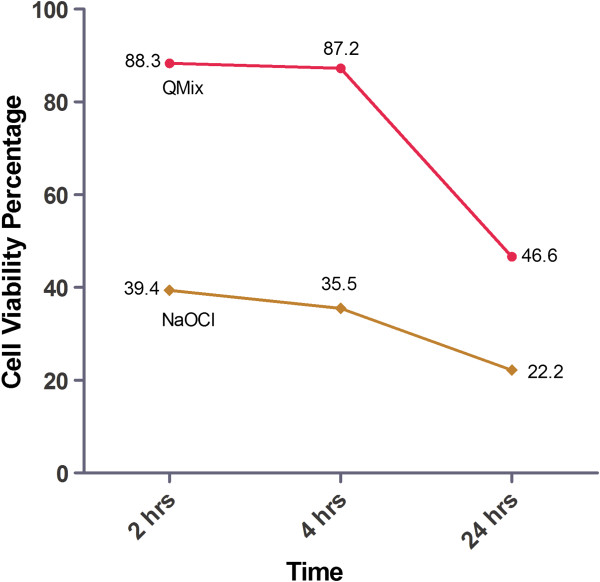
The cell viability percentage for NaOCl and QMix solutions at 2, 4 and 24 hours after exposure using MTT assay.

The MTT assay was conducted on three groups (the QMix™ group, NaOCl group, and Control group) with cultured hTERT-MSCs in 96-well plates. Each group was incubated for 2, 4 and 24 hours. The cell viability for each group is presented as percentage of the control group at each time point. The percentage cell viability of each solution is illustrated in Figure 1. When the cells were exposed to solutions for 2, 4 and 24 hours, the cell viability decreased with time. The viability of cells exposed NaOCl was significantly lower than that of cells exposed to QMix™ at all time intervals (P < 0.05).

### AlamarBlue assay

**Figure 2:**
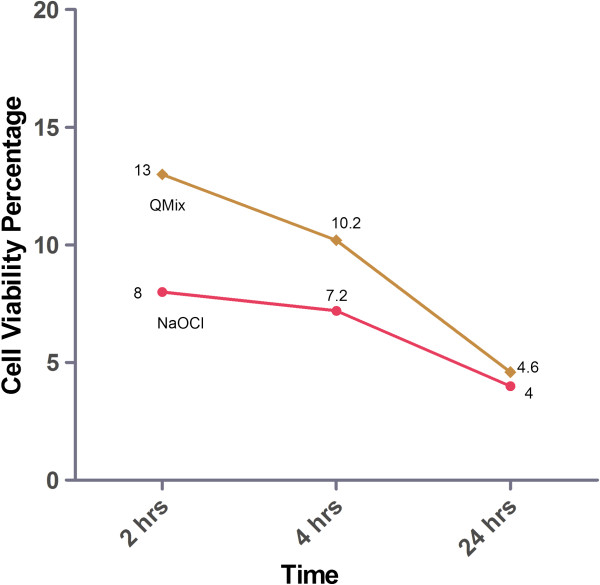
The cell viability for both NaOcl and QMix™ solutions using alamarBlue assay at 2, 4 and 24 hours of exposure.

The AB assay was conducted for the QMix™, NaOCl and Control groups using cultured hTERT-MSCs in 96-well plates. The cell viability for each group is presented as the percentage of the control group. The cell viability percentage of each solution is illustrated in Figure 2. When the cells were incubated for 2 or 4 hrs, the cell viability of the NaOCl group was significantly lower than that of the QMix™ group (P < 0.05).

### Cell morphology

**Figure 3:**
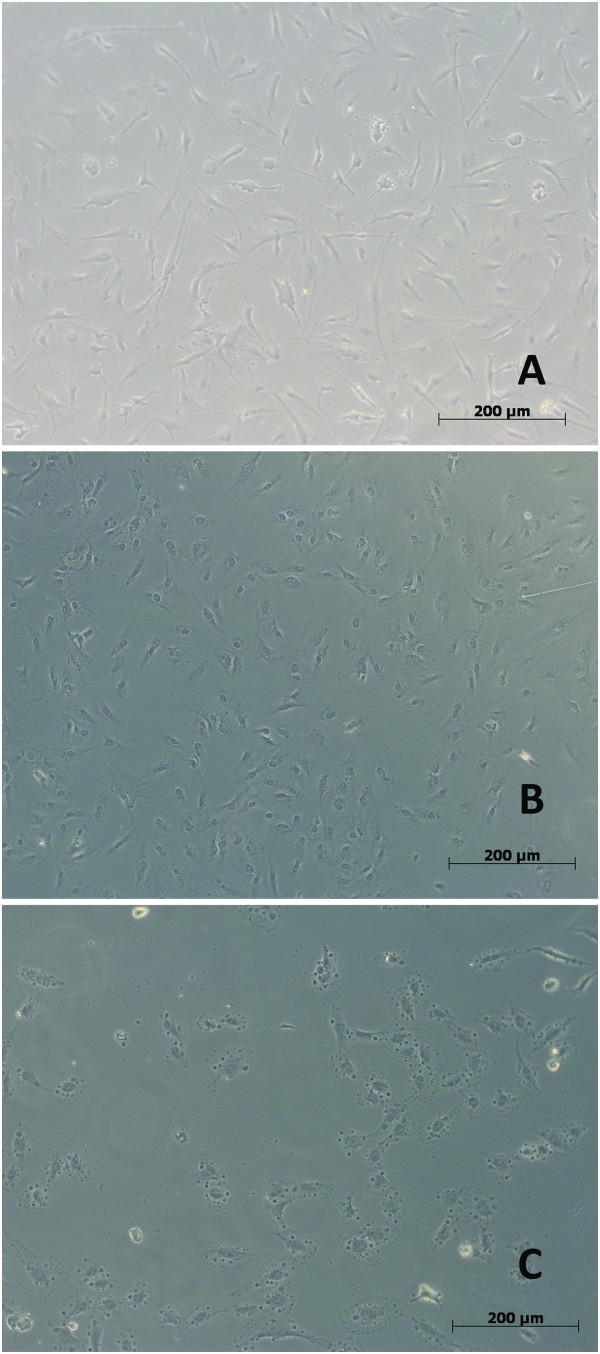
**Human bone marrow hTERT-MSCs under inverted LM after adding the testing agents (X10). A**- Untreated cells, **B** - Cells exposed to (0.5) Qmix™ solution (the cell shape was maintained with few alterations), **C**- Cells exposed to (0.5) NaOCl; vacuolization was evident in the cytoplasm.

Untreated negative control cells showed variable shapes, including rhomboid, triangular, oval and spindle shapes, as illustrated by light microscopy LM (Figure 3) Cells were attached to each other and to the substrate. The cell wall appeared smooth and intact, with some microvilli and surface extensions. According to the LM investigation, the toxic effect of the test solutions was evident immediately after their application to the cultured human bone marrow MSCs, and the normal cell morphology was altered differently in each group (Figure 3). A high concentration (0.5 mg/ml) of NaOCl caused the vacuolization of the cytoplasm (Figure 3B), whereas QMix™ at the same concentration produced few alterations in the cell wall and no vacuolization (Figure 3C).

**Figure 4:**
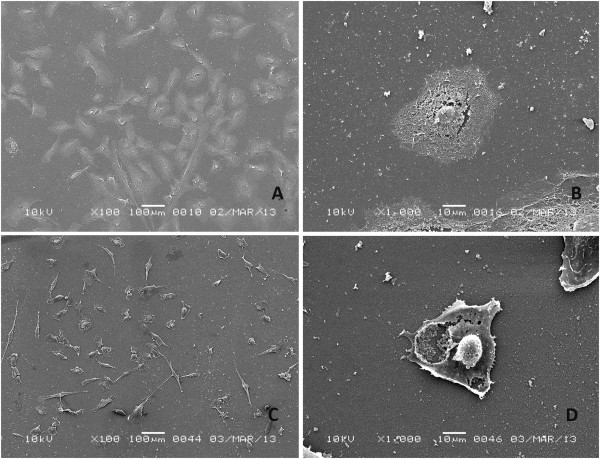
**Human bone marrow hTERT-MSCs exposed for 2 hrs to QMix and NaOCl. A** - QMix™ (x100), **B** - QMix™ (x1000), **C** - NaOCl (x100, note the round or thread-like shape of the remnant cells), **D**- NaOCl (x1000, the ruptured cell wall and the extruded nucleus).

According to the SEM analysis, the cell number decreased relative to the control after a 2-hour exposure to 0.5 mg/ml NaOCl. The remaining cells were smaller, with a thread-like or round shape (Figure 4). Cells became detached from the subsurface, and cell-to-cell attachments were lost. Lysosomal secretions emerging from the cell were observed, and the nucleus was extruded through the ruptured cell wall.

**Figure 5:**
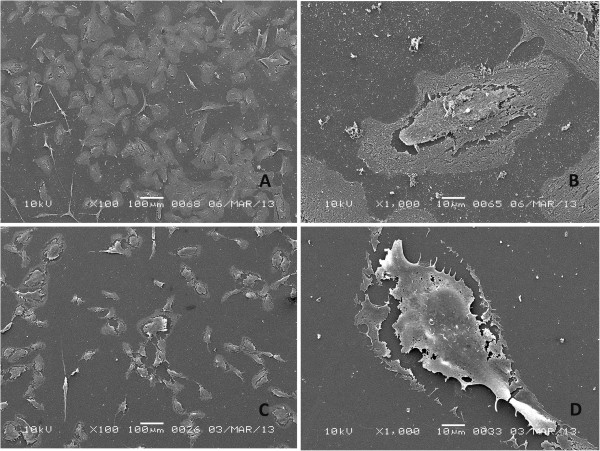
**Human bone marrow hTERT-MSCs exposed for 4 hrs to QMix™ and NaOCl. A**- QMix™ (x100), **B**-QMix™ (x1000), **C** - NaOCl (x100), **D**- NaOCl (x1000).

After a 2-hour exposure to QMix™, the cell number and cell shape were similar to those of the control, except for the presence of an ill-defined cell outline. However, the cells were still attached to adjacent cells and to the substrate (Figure 4). In some areas, remnants of cell processes were observed. The main dramatic change in the cell morphology was in the cell wall, which had a mesh-like appearance (Figure 4B), indicating disintegration. After 4 hours, the alteration of cell morphology was more significant: the mesh-like appearance of the cells became more intense, with an ill-defined shape and a fading outline, and broken cell-to-cell attachments were observed. However, the attachment to the substrate was maintained by some cells (Figure 5).

### Live/dead analysis with EB/AO staining

**Figure 6:**
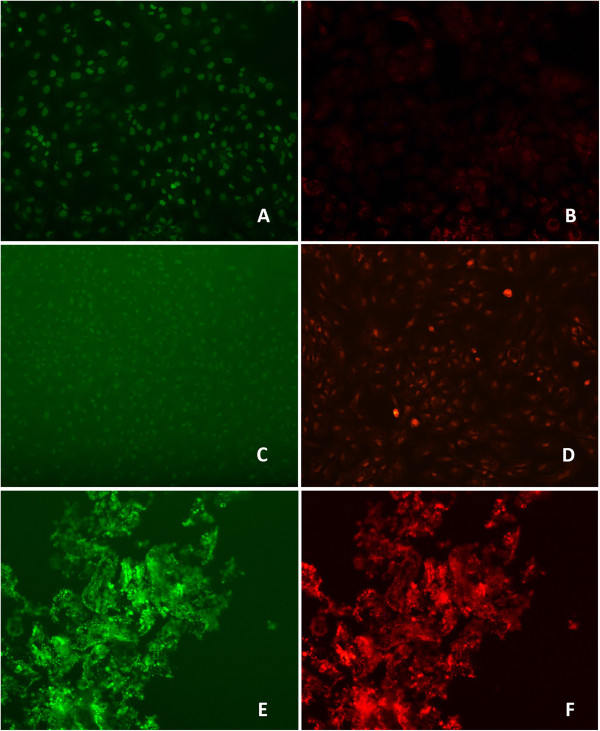
**Live/dead analysis human bone marrow hTERT-MSCs under a fluorescence microscope (X10).** 6**A**, 6**B** – Untreated cells with the green filter, the nucleus appears intact , which indicates a viable cell. 6**B**-with the red filter, the nucleus appeared dark, and only the external surface of the cells was red, indicating that the cell wall is intact. 6**C**, 6**D**- treated with QMix™ under a fluorescence microscope (X10), 6E,6F- treated with NaOCl under a fluorescence microscope (X10).

Cultured human bone marrow hTERT-MSCs were exposed to 0.5 mg/ml of QMix™ and NaOCl for 2 hrs. The EB/AO stain was applied to the cells, which were then examined under an inverted fluorescence microscope. The images for each group display live cells in green and dead cells in red. The untreated cells had intact, well-defined nuclei, which were green under the green filter, as shown in Figure 6A. Under the red filter, only the cell surface showed red fluorescence, not the nucleus, which indicated an intact cell wall (Figure 6B). When the cells were exposed to QMix™, the cell number was similar to that of the control group, and the nuclei were green, which was similar to the appearance of the control cells. The only difference was that some of these cells had condensed chromatin, which was evident under the red filter (Figure 6C, D). When the cells were exposed to NaOCl, no cell structures, such as the nucleus or cell membrane, were observed; cell lysis was evident, and the remnant material was positive for both green and red fluorescence (Figure 6E, F).

## Discussion

This *in vitro* study was conducted to assess the cytotoxicity of the QMix™ irrigating solution on human bone marrow MSCs. MSCs have been suggested as a good model for toxicological testing [29]. The MSCs that were used in this study were immortalized by the ectopic expression of human telomerase reverse transcriptase (h-TERT), which increased the life span of the cells [24] and maintained their stem-like properties [30]. Earlier studies have reported that immortalized cells can be used as a test model for dental materials [31].

The observations from the study showed that both solutions (QMix™ and NaOCl) are toxic to human bone marrow MSCs and cause cellular damage. This is consistent with the results of previous studies that reported on NaOCl toxicity [23,32,33]. NaOCl toxicity can be attributed to its high pH (hydroxyl ion action), which interferes with cytoplasmic membrane integrity [34]. Furthermore, our results are in agreement with those of a previous *in vivo* study, which found that QMix™ is toxic and can induce an inflammatory response [35]. CHX is a toxic agent that binds to the cell’s plasma membrane and increases its permeability, allowing the leakage of lysosomal enzymes [36]. EDTA, which is the second QMix™ component, is also known to be cytotoxic, perhaps due to its chelating effect and the accentuated drop in pH that it causes [11].

Cell viability decreased significantly when the cells were exposed to NaOCl for all time periods examined. Cell viability decreased significantly after being exposed to the QMix™ solution for 2 or 4 hours. Moreover, after 24 hours, cell viability was significantly decreased compared to 2 and 4 hours of exposure. These findings show that the toxic effect of an agent gradually increases with time. This observation is in agreement with those of previous studies, confirming that toxicity is time dependent [37]. In contrast, the MTT assay results showed a significant decrease in the cell viability of cells that were exposed to NaOCl at all time periods examined. Compared with QMix™-exposed cells, NaOCl decreased viability at 2 and 4 hours .

Previous studies have reported that the AB assay is slightly more sensitive than the MTT assay. However, both assays rely on enzymatic metabolism, which may be inhibited or induced by the testing agent, thus producing a false-positive or false-negative result. Therefore, careful interpretation of the results is always recommended [38]. Our observations suggest that the AB assay is a better choice for cell viability testing because it is easy to perform, more consistent than the MTT assay, and recommended by previous studies [38,39]. However, it is always recommended to use more than one assay to assess cytotoxicity. Therefore, previous studies that relied solely on the MTT assay should be re-evaluated and interpreted with caution.

Microscopic morphological investigations are necessary to confirm cellular toxicity [40]. This is because a cell can undergo toxic changes, such as detachment, while still continuing to metabolize MTT to formazan, resulting in an over estimation of cell viability in the MTT assay compared with the AB assay [38]. Therefore, cellular morphological characteristics were investigated for both solutions.

Cells that were exposed to QMix™ displayed fewer morphological alterations. This observation is in agreement with Faria et al., who reported that higher concentrations of CHX would preserve the shape of the L929 cells [41] due to its cell fixation effect [42].

The SEM images for the QMix™ group showed cytoplasmic shrinkage with partially fragmented but intact cell walls. These characteristics are typical of apoptosis, as reported previously [40]. In addition, the EB/AO staining showed bright green fragmented nuclei, indicating early apoptosis [27]. The cells exposed to NaOCl had cytoplasmic shrinkage or ruptured membranes, which are typical characteristics of necrosis [43]. The EB/AO staining of the NaOCl group displayed red and green, reflecting remnant material that may be the result of cell lysis, and no cell structure could be observed. The overall analysis of these results suggests that the cells in the QMix™ groups are in the early stages of apoptosis but are not yet dead, in contrast to the NaOCl group. Both NaOCl and QMix™ are cytotoxic; however, it seems that their mode of cell killing differs as a result of their different compositions. According to Galluzzi et al. [44], cell death can be classified into four different types based on the morphological characteristics of the dying cells: apoptosis (Type 1), autophagy (Type 2), necrosis (oncosis, Type 3), and mitotic catastrophe. Each mode of cell death has its own function; necrosis induces an inflammatory response when it is needed, whereas apoptotic cells *in vivo* are rapidly phagocytosed without inducing an inflammatory response, which is considered a mechanism for avoiding immune activation. According to our findings, this mode of cell death could be associated with a high concentration exposure to NaOCl.

Apoptosis is an active form of cell death known as programmed cell death, and it is characterized by cell shrinkage and the nuclear chromatin condensation followed by nuclear fragmentation, with the normal morphological appearance of cytoplasmic organelles and the maintenance of an intact plasma membrane [44]. According to our findings, this mode of cell death could be associated with QMix™ exposure. However further markers such as detection of caspases, cleaved substrates, regulators and inhibitors are necessary to substantiate this mode of cell death speculated with QMix™ [45]. Cell death through apoptosis is known to occur through two primary pathways: an extrinsic pathway that involves death receptors, and an intrinsic pathway that is modulated by members of the Bcl-2 family [46], which consists of outer mitochondrial membrane components [47]. Therefore, mitochondria are considered key organelles in the pathways to cell death in addition to its normal metabolic activity [48,49]. However, these proteins may also function in some normal metabolic pathways. Thus, mitochondrial metabolic activity investigations, such as the MTT assay, must consider these potential overlaps between pre-apoptotic cell activity and normal cell metabolism [40]. This overlap could explain the contradictory results of the AB and MTT assays.

*In vitro* cytotoxicity investigations reported that CHX had a higher toxicity in cell cultures than NaOCl [33,41]. However, *in vivo* studies suggest that CHX or QMix™ is less aggressive than NaOCl [35,50]. This difference could be attributed to host defense mechanisms that operate in the *in vivo* environment.

Our *in vitro* study has the following limitations: it was conducted on cultured cells, and the results represent only the response of these cells in isolation, without taking into account the host defense mechanism for detoxification. Furthermore, in a clinical setting, the solutions are always delivered to root canals, which are surrounded by dentine, and are then extruded to the periapical area. Previous studies have reported that the cytotoxic effect of irrigants can be neutralized by dentine [33,51]. We placed the solutions directly onto the cells, and no attempts were made to deliver these solutions through root canals to mimic the clinical scenario.

## Conclusions

Within the limitations of this study, it can be concluded that both the NaOCl and QMix™ solutions are toxic to human bone marrow MSCs. The QMix™ solution, which induces slow cell death, seems to be more biocompatible than the NaOCl solution. Hence, based on these observations, it can be concluded that the QMix™ is a relatively safer root canal irrigant compared to NaOCl.

## Competing interests

The authors declare that they have no competing interests.

## Authors’ contributions

AK, SMA, AM and MAE carried out the study, laboratory procedures and evaluation of the results. SA, AK and AMA involved in the development of the concept, design of the study, revision of the manuscript and statistical analysis. All authors read and approved the final manuscript.

## Pre-publication history

The pre-publication history for this paper can be accessed here:

http://www.biomedcentral.com/1472-6831/14/27/prepub
